# Afatinib-Induced Tumor Lysis Syndrome in Pulmonary Adenocarcinoma: A Case Report and Literature Review

**DOI:** 10.3390/medicina59122144

**Published:** 2023-12-10

**Authors:** Goohyeon Hong

**Affiliations:** Division of Pulmonary and Critical Care Medicine, Department of Internal Medicine, Dankook University Hospital, Dankook University College of Medicine, 201 Manghyang-ro, Dongnam-gu, Cheonan 31116, Republic of Korea; hkh0519@hanmail.net; Tel.: +82-41-550-3953; Fax: +82-41-559-7940

**Keywords:** pulmonary adenocarcinoma, tumor lysis syndrome, targeted therapy, afatinib

## Abstract

Tumor lysis syndrome (TLS) is a potentially fatal oncological emergency that typically develops during the treatment of rapidly proliferating malignancies. It is infrequently reported in solid tumors, such as pulmonary adenocarcinoma. A 59-year-old male patient with shortness of breath presented with a 3.3 cm × 3.0 cm mass in the right upper lobe, along with massive right-sided pleural effusion. A percutaneous needle biopsy was performed, and a diagnosis of pulmonary adenocarcinoma with an epidermal growth factor receptor (*EGFR*) mutation was made. The patient was treated with afatinib because of the malignant pleural effusion and multiple metastases to the intrathoracic lymph nodes, left scapula, and brain. After 4 days of afatinib treatment, he developed oliguric acute kidney injury and progressively worsening dyspnea. Based on the clinical and laboratory findings, the patient was diagnosed with afatinib-induced TLS. To the best of our knowledge, this is the first reported case of afatinib-induced TLS in pulmonary adenocarcinoma.

## 1. Introduction

Tumor lysis syndrome (TLS) is a life-threatening oncological emergency that typically results from the lysis of rapidly proliferating malignant cells with a high tumor burden, such as hematological malignancies after the initiation of cytotoxic chemotherapy [1,2,3,4]. The most common hematological malignancies associated with TLS are acute leukemias and high-grade non-Hodgkin’s lymphoma, particularly Burkitt lymphoma. Although TLS is rare in patients with solid tumors [5], there have been few reports of TLS in patients with lung cancer [6,7,8]. Most reports have described patients with small cell lung cancer (SCLC), whereas some reports have described patients with non-small cell lung cancer (NSCLC). Additionally, there have been few reports of pulmonary adenocarcinoma treatment with targeted agents, including epidermal growth factor receptor tyrosine kinase inhibitor (EGFR-TKI) and anaplastic lymphoma kinase tyrosine kinase inhibitor (ALK-TKI) [9,10].

Here, we report a case of acute TLS in a patient with pulmonary adenocarcinoma and multiple metastasis who received first-line chemotherapy with afatinib, an EGFR-TKI.

## 2. Case Report

A 59-year-old male patient with a known 30 pack-year smoking history was admitted with shortness of breath for 1 week. He was previously healthy with no significant past medical history of hypertension, diabetes, hepatitis, chronic lung disease, or malignancy. Upon admission, his body mass index was 23.88 (height 165 cm, weight 65 kg) and vital signs included a blood pressure of 146/110 mmHg, body temperature of 36.8 °C, heart rate of 120 beats/min, and respiratory rate of 25 breaths/min. A chest auscultation revealed decreased breath sounds in the right lung. An arterial blood gas analysis revealed a pH of 7.41, pCO_2_ of 30.0 mmHg, PaO_2_ of 64.0 mmHg, HCO_3_ of 19.0 mmol/L, and SpO_2_ of 92% while receiving oxygen through a nasal prong at a flow rate of 2 L/min. The complete blood count results were as follows: leukocytes, 11,410/mm^3^ (segmented neutrophils 74%, lymphocytes 13.8%, monocytes 10.8%); hemoglobin, 16.7 g/dL; hematocrit, 49.2% and platelets, 394,000/mm^3^. The blood chemistry was as follows: blood urea nitrogen (BUN), 15.0 mg/dL; creatinine, 0.96 mg/dL; aspartate transaminase, 28 U/L; alanine transaminase, 27 U/L; total bilirubin, 1.2 mg/dL; creatine kinase, 62 U/L; lactate dehydrogenase, 247 U/L; uric acid, 4.5 mg/dL; calcium, 8.6 mg/dL; phosphorous, 2.5 mg/dL. The prothrombin time (PT), international normalized ratio (INR), and activated partial thromboplastin time (aPTT) were 13.1, 1.2, and 30.4 s, respectively.

**Figure 1:**
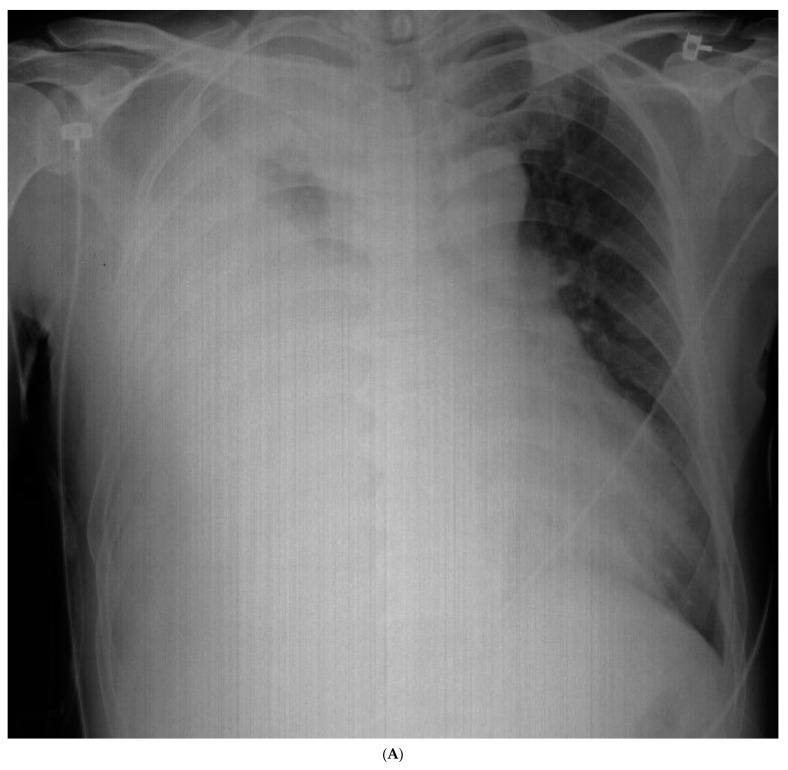

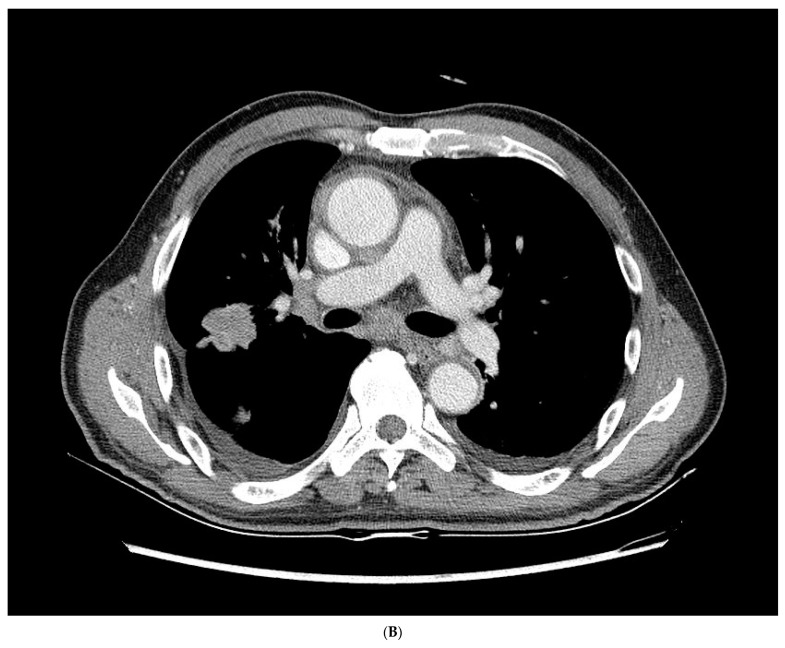
Initial chest radiography and post-drainage chest computed tomography showing a right-sided pleural effusion. (**A**) Chest radiography revealed a massive right-sided pleural effusion. (**B**) After drainage of the right-sided pleural effusion, chest computed tomography showed a 3.3 cm × 3.0 cm mass in the right upper lobe.

**Figure 2:**
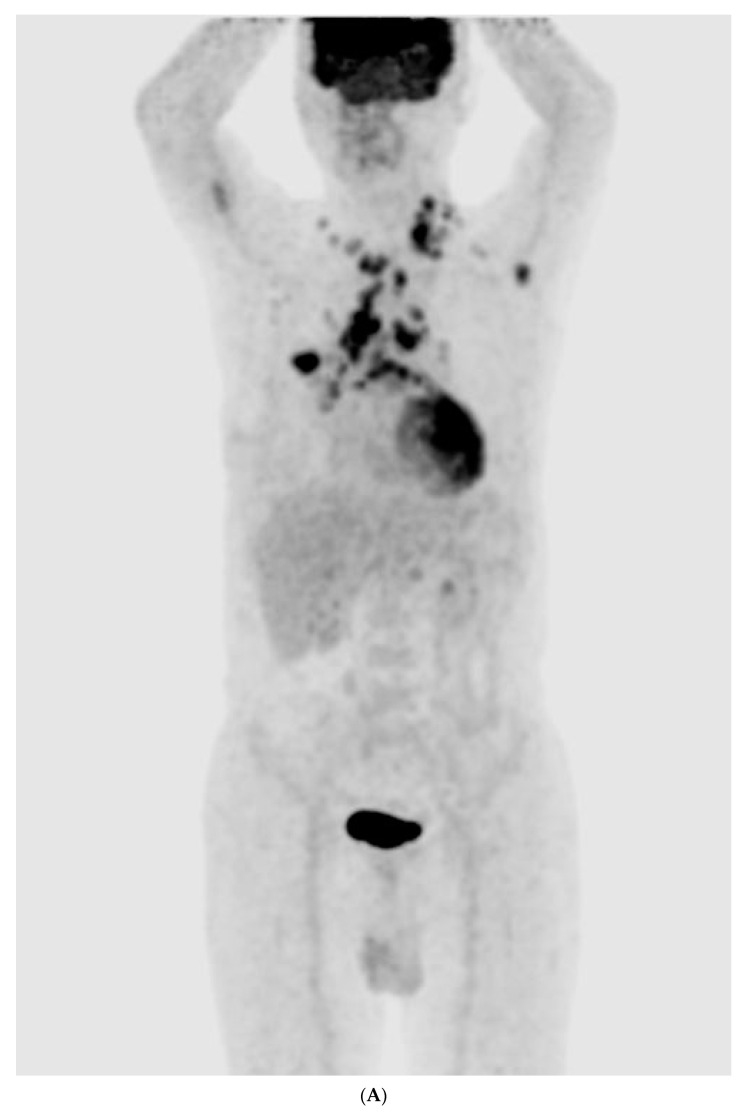

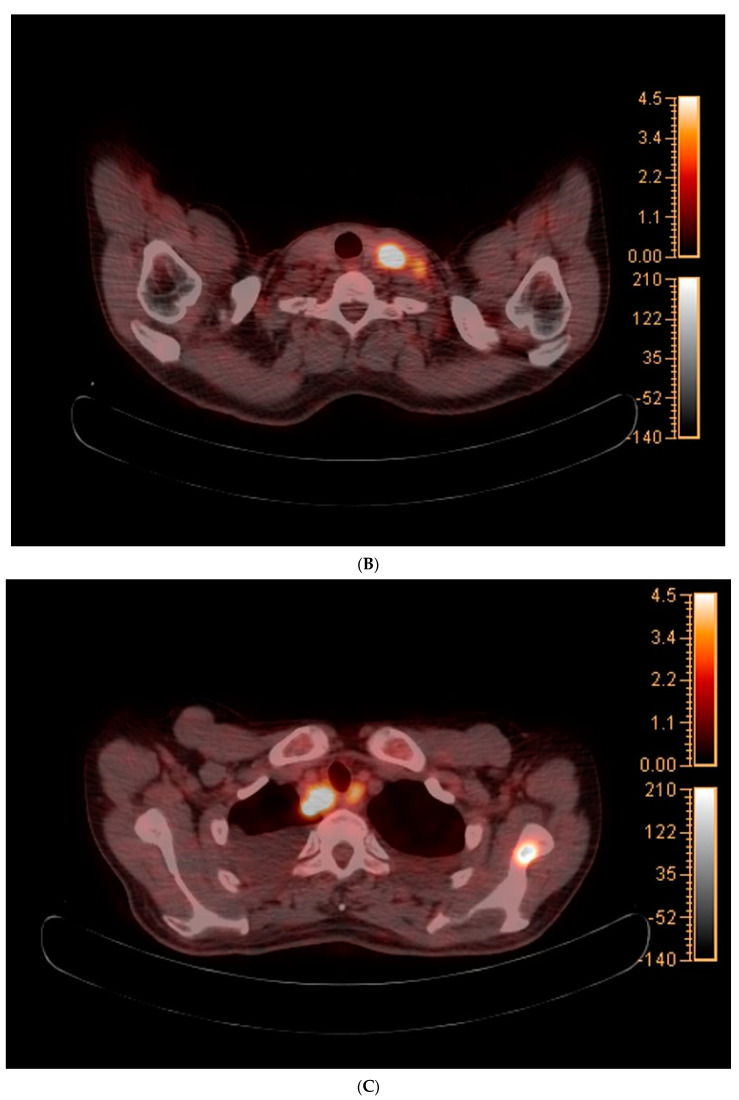

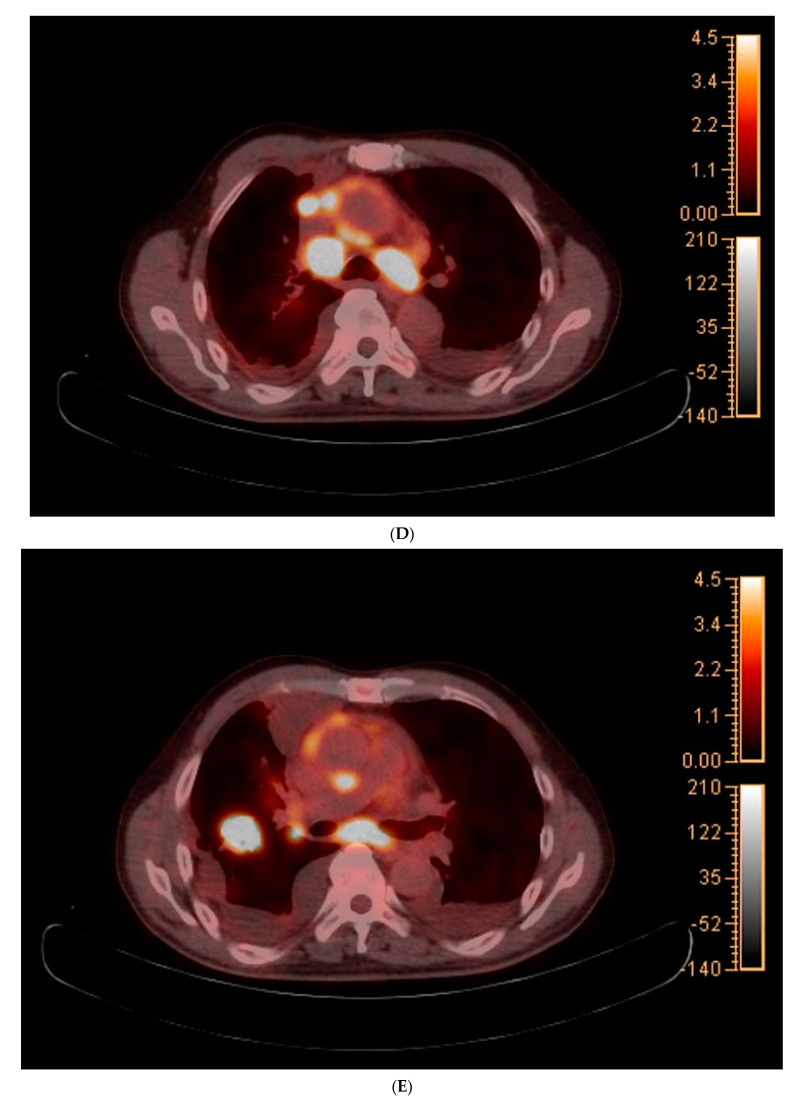
(**A**–**E**) Positron emission tomography/computed tomography revealed metastases to multiple lymph nodes, includingleft supraclavicular lymph node, bilateral mediastinal areas, and left scapula.

**Figure 3:**
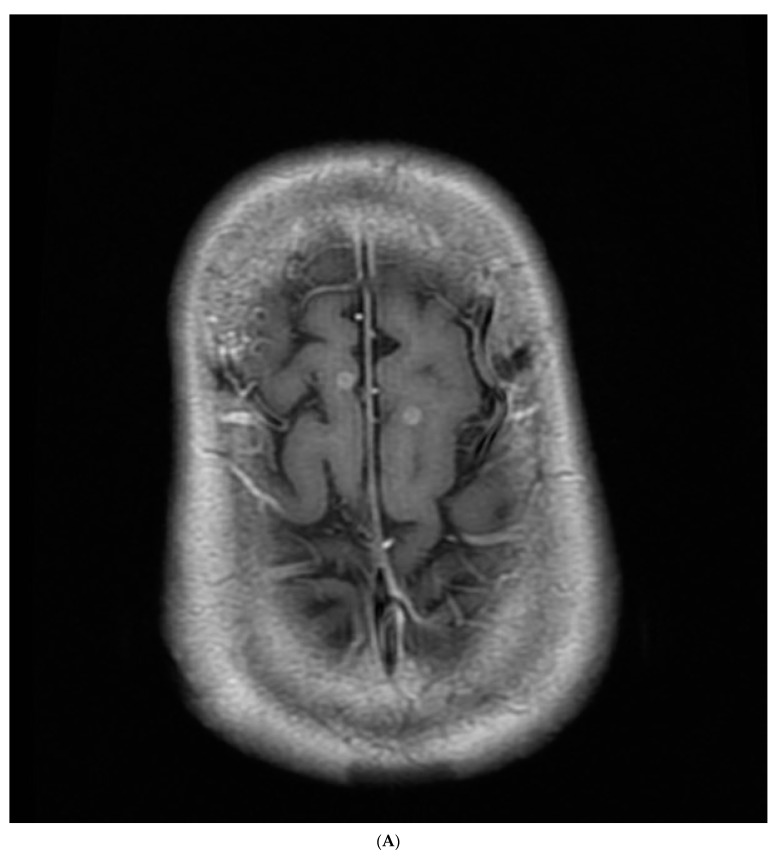

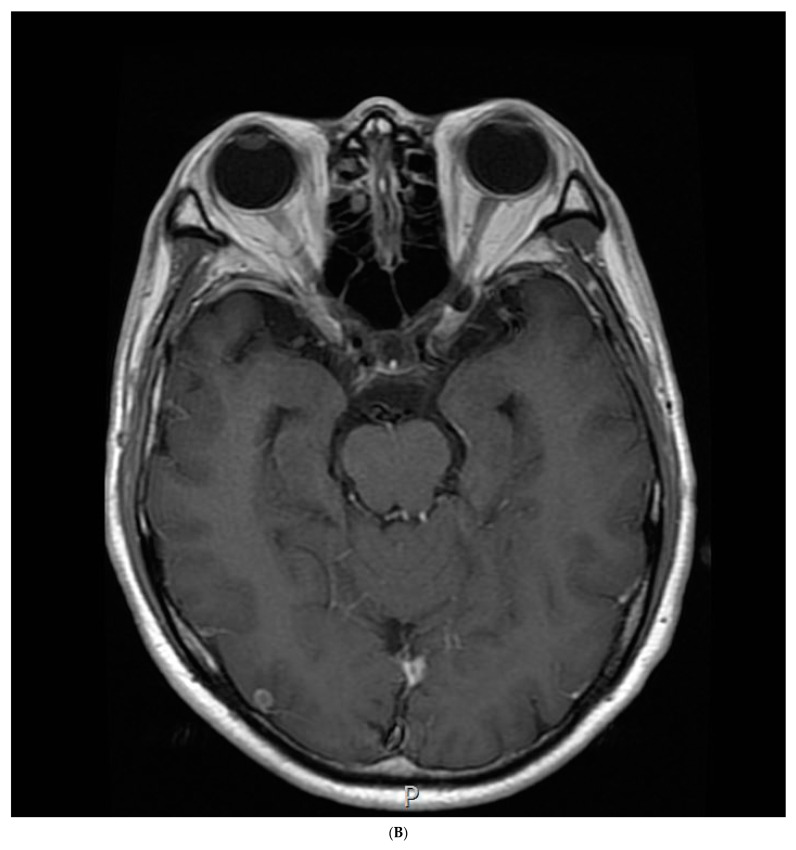

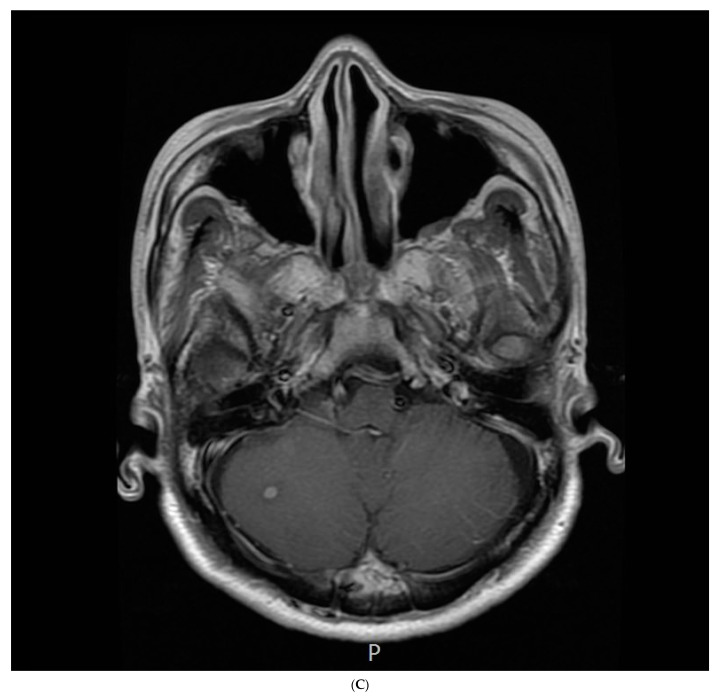
Magnetic resonance imaging of the brain revealed (**A**,**B**) multiple tiny cerebral and (**C**) right cerebellar metastases.

The chest radiography showed a massive right-sided pleural effusion (Figure 1A). Percutaneous catheter drainage was performed for the right-sided pleural effusion. The chest computed tomography showed a 3.3 cm × 3.0 cm mass in the right upper lobe (Figure 1B). The positron emission tomography/computed tomography revealed metastases in the left-sided supraclavicular lymph nodes, multiple bilateral mediastinal lymph nodes, and left scapula (Figure 2). The magnetic resonance imaging of the brain revealed multiple tiny cerebral and right cerebellar metastases (Figure 3). A percutaneous needle biopsy confirmed the diagnosis of lung adenocarcinoma (pT2aN3M1c, stage IVB); the analysis of the right-sided pleural effusion revealed metastatic adenocarcinoma. The genetic analysis revealed an exon 19 deletion in the *EGFR* gene. The laboratory tests, including the blood cell counts and chemistry, were within the normal ranges prior to treatment initiation. The patient was administered the EGFR-TKI afatinib (40 mg/day) as a first-line chemotherapy.

On the fourth day of afatinib administration, the patient’s urine output decreased and dyspnea worsened. The laboratory tests revealed the following results: BUN, 55 mg/dL; creatinine, 2.2 mg/dL; potassium, 5.9 mmol/L; phosphorus, 7.0 mg/dL; calcium, 8.0 mg/dL; lactate dehydrogenase, 1082 U/L; and uric acid, 10.0 mg/dL. Based on the clinical manifestations and laboratory findings, a diagnosis of afatinib-induced TLS was established. Despite the discontinuation of afatinib, intensive supportive management (i.e., vigorous hydration with saline, concomitant use of furosemide, and urinary alkalinization with sodium bicarbonate), and specific interventions for hyperuricemia and hyperkalemia, the patient’s clinical parameters did not improve and his laboratory parameters further deteriorated (blood urea nitrogen, 63 mg/dL; creatinine, 3.07 mg/dL; uric acid, 12.0 mg/dL; phosphorus, 9.6 mg/dL; and potassium, 6.1 mmol/L). The laboratory abnormalities associated with TLS at different time points are shown in Table 1. The patient was administered continuous renal replacement therapy for the TLS. However, his condition rapidly deteriorated; unfortunately, he died the next day.

## 3. Discussion

Lung cancer is one of the most common cancers worldwide and a leading cause of cancer-related deaths, with an estimated mortality rate of >1 million per year. NSCLC causes approximately 85% of lung cancer cases [11]. Because of the advancements in molecular analysis and targeted therapies, molecular TKIs have become the standard treatment for patients with advanced-stage NSCLC harboring targetable mutations. The most common therapeutic targets in NSCLC include *EGFR* and *ALK* alterations [12]. EGFR-TKIs are widely used and recommended as a first-line treatment in patients with metastatic pulmonary adenocarcinoma harboring *EGFR* mutations [13,14]. Afatinib is a second-generation EGFR-TKI that is useful for the treatment of *EGFR* mutation-positive pulmonary adenocarcinoma. Afatinib is administered orally, at a recommended dose of 40 mg once daily and can be considered without the need for a starting dose adjustment in patients with mild or moderate renal impairment and mild or moderate hepatic impairment, although a starting dose of 30 mg once daily is recommended in patients with severe renal impairment. In cases with severe hepatic impairment, afatinib has not been studied. Therefore, these patients should be closely monitored and the dose adjusted, if needed. The most common afatinib-related adverse events are diarrhea, decreased appetite, and skin reactions including rash, acne, stomatitis, itchiness, and dry skin [15].

TLS is common in hematological malignancies but uncommon in solid tumors because of their relatively low proliferative rate and limited response to chemotherapy. Hepatocellular carcinoma, SCLC, malignant melanoma, breast cancer, and germ cell tumors are the solid tumors most commonly associated with TLS [4,7,8]. TLS is rare in patients with NSCLC. Although several cases of TLS have been identified in patients with pulmonary adenocarcinoma, most of these cases were induced by cytotoxic chemotherapy (Table 2).

The occurrence of TLS in association with targeted therapy is very rare. Furthermore, few cases of TLS have been identified in patients with pulmonary adenocarcinoma receiving targeted agents, such as EGFR-TKIs and ALK-TKIs [9,10]. Only two cases of TLS have been identified in patients with pulmonary adenocarcinoma receiving targeted therapy. The first case was induced by gefitinib, a first-generation EGFR-TKI [9]; the other case was associated with brigatinib, an ALK-TKI [10]. To the best of our knowledge, this is the first case of afatinib-induced TLS in pulmonary adenocarcinoma.

The most commonly used diagnostic criteria for TLS were proposed by Cairo and Bishop in 2004 [2]. The criteria categorize the disease as laboratory TLS or clinical TLS. Laboratory TLS is diagnosed when two or more of the following laboratory abnormalities develop within 3 days before or up to 7 days after the initiation of therapy: hyperuricemia, hyperphosphatemia, hyperkalemia, and hypocalcemia. Clinical TLS is defined as the presence of laboratory TLS and one or more of the following complications: renal insufficiency, seizures, and cardiac arrhythmias or sudden death [2]. After 4 days of afatinib treatment, our patient exhibited oliguric acute kidney injury and characteristic laboratory abnormalities, including hyperkalemia, hyperuricemia, hyperphosphatemia, and hypocalcemia. The patient’s clinical and laboratory findings fulfilled the diagnostic criteria for TLS. Consequently, he was diagnosed with afatinib-induced TLS.

We have identified seven previous reports of the occurrence of TLS in patients with pulmonary adenocarcinoma [9,10,16,17,18,19,20]. All seven patients had metastatic disease: four had liver metastases and four had lymph node involvement. These findings suggest that patients with advanced metastatic pulmonary adenocarcinoma have a high risk of TLS. In the present case, the patient had lymph node, pleural, bone, and brain metastases.

Several risk factors can predict the development of treatment-related TLS, including highly proliferative and bulky malignancies with metastases; high sensitivity to chemotherapy; preexisting renal insufficiency; exposure to nephrotoxic substances; underlying problems (e.g., infections and intravascular volume depletion); and baseline elevations in uric acid, phosphorus, and lactate dehydrogenase levels [1,2]. These factors should be used to identify high-risk patients who require interventions for TLS prevention. The mainstay of management for TLS involves aggressive hydration and the use of diuretics and uric acid-lowering agents. Patients who have low risk for TLS can be managed with allopurinol; rasburicase treatment should be considered for high-risk patients. However, glucose-6 phosphate dehydrogenase deficiency must be excluded before rasburicase is administered because this medication can cause severe hemolysis [21,22,23]. Dialysis or renal replacement therapy can be considered in patients with refractory hyperkalemia secondary to oliguria and symptomatic hypocalcemia related to hyperphosphatemia. Continuous renal replacement therapy is preferred in the presence of TLS because it removes phosphate more efficiently, compared with regular hemodialysis [21,22,23,24].

The mortality rate is higher for patients with solid tumors and TLS than for patients with hematological malignancies and TLS. Among the seven previously reported cases of TLS in pulmonary adenocarcinoma, six cases resulted in death, despite aggressive management. The high mortality rate in solid tumor patients with TLS may be explained by the non-administration of preventive measures for TLS; such measures are routinely administered prior to chemotherapy for hematological malignancies [6,7,8]. Unlike TLS in hematological malignancies, TLS in solid tumors can develop after days to weeks of treatment. Additionally, NSCLC patients are typically treated with oral molecular TKIs at home, rather than at the hospital. Although rare, TLS can develop in pulmonary adenocarcinoma treated with targeted agents, including EGFR-TKIs and ALK-TKIs. Thus, patients with such tumors require risk stratification, careful observation, and laboratory testing during the initial phase of targeted therapy.

Our report highlights the risk of TLS during treatment with targeted agents, including EGFR-TKIs and ALK-TKIs, in pulmonary adenocarcinoma. Physicians should be aware of this rare but potentially fatal complication in patients with pulmonary adenocarcinoma who have a high risk of TLS.

## 4. Conclusions

To the best of our knowledge, this is the first reported case of afatinib-induced TLS in pulmonary adenocarcinoma. TLS is an oncological emergency that can be fatal; therefore, the early recognition of risk factors, close monitoring of high-risk patients, and effective interventions are necessary for patients with pulmonary adenocarcinoma receiving targeted agents.

## Figures and Tables

**Table 1:** Laboratory parameters of the patient at different time point.

Parameters	Reference Range	Day of Admission	4th Day Morning of Afatinib Administration	4th Day Evening of Afatinib Administration	During CRRT (5th Day of Afatinib Administration)
BUN (mg/dL)	6–20	15.0	55.0	63	30.0
Creatinine (mg/dL)	0.7–1.2	0.96	2.2	3.07	2.57
Potassium (mmol/L)	3.5–5.5	4.3	5.9	6.1	4.4
Phosphorous (mg/dL)	2.7–4.5	2.5	7.0	9.6	4.4
Calcium (mg/dL)	8.4–10.4	8.6	8.0	7.7	7.7
Uric acid (mg/dL)	3.4–7.0	4.5	10.0	12	8.2
LDH (U/L)	135–250	247	1082	8202	8113

BUN, blood urea nitrogen; LDH, lactate dehydrogenase; CRRT, continuous renal replacement therapy.

**Table 2:** Previous reports of tumor lysis syndrome in patients with pulmonary adenocarcinoma.

Case	Sex/Age	Chemotherapy	Metastasis	Onset Interval	Outcome
Persons [16]	F/38	Irinotecan + cisplatin	Lymph nodes	13 days	Survived
Feld [17]	M/72	Spontaneous	Liver	N/A	Died
Kurt [18]	M/52	Zoledronic acid	Brain, liver, paraspinal, bones, and small intestine	4 days	Died
Ajzensztejn [19]	M/65	Docetaxel	Liver, kidneys, and adrenals	3 days	Died
Honda [20]	M/61	Carboplatin + paclitaxel + bevacizumab	Liver and lymph nodes	N/A	Died
Eguchi [9]	M/73	Gefitinib	Bones and lymph nodes	5 days	Died
Wang [10]	M/39	Brigatinib	Pleura and lymph nodes	22 days	Died

N/A, not applicable.

## Data Availability

The data of this study are available from the corresponding author upon reasonable request.
